# Supplementary material for: RadPhysBio: A Radiobiological Database for the Prediction of Cell Survival upon Exposure to Ionizing Radiation
Source: Int J Mol Sci. 2024 Apr 26;25(9):4729. doi: 10.3390/ijms25094729 (PMC11083482; doi:10.3390/ijms25094729)
Supplement: Supplementary file 1 [file ijms-25-04729-s001.zip › ijms-2844178-supplementary.pdf]

SUPPLEMENTARY DATA

RadPhysBio: a radiobiological database for the prediction of cell survival upon exposure to ionizing radiations

Vassiliki Zanni[1], Dimitris Papakonstantinou [2], Spyridon A Kalospyros[1], Dimitris Karaoulanis[3], Gökay Mehmet Biz[4], Lorenzo Manti[5], Adam Adamopoulos[6], Athanasia Pavlopoulou[4,7] and Alexandros G Georgakilas [1,*]

[1] DNA Damage Laboratory, Physics Department, School of Applied Mathematical and Physical Sciences, National Technical University of Athens (NTUA), Zografou Campous, 15780 Athens, Greece; fte17010@mail.ntua.gr ; spkals@central.ntua.gr
[2] Department of Life Sciences, University Paris-Saclay, 91190 Saint-Aubin, Paris, France; dimitrispapak@gmail.com
[3] School of Electrical and Computer Engineering, National Technical University of Athens, 15780 Athens, Greece; dkaraoul@gmail.com
[4] Izmir Biomedicine and Genome Center (IBG), Balcova, Izmir 35340, Turkey; gkymhmt@gmail.com
[5] National Institute of Nuclear Physics (INFN), Section of Naples, Naples Italy and Radiation Biophysics Laboratory, Department of Physics "E. Pancini", University of Naples Federico II, Naples, Italy; manti@na.infn.it
[6] Department of Medicine, Medical Physics Laboratory, Democritus University of Thrace, 681 00 Alexandroupolis, Greece; adam@med.duth.gr
[7] Izmir International Biomedicine and Genome Institute, Dokuz Eylül University, 35340 Balcova, Izmir, Turkey; athanasiapavlo@deu.edu.tr
*Correspondence: alexg@mail.ntua.gr; Tel.: +30-210-7724453

The Python code for the calculation of α and β coefficients included in the database

```
import numpy as np

from scipy.optimize import curve_fit

points = np.array([(4.0114,0.2176), (6.0114,0.11083), (8.0114,0.077946)])

xdata = points[:,0]

ydata = points[:,1]

def f(x,a,b):

    return np.exp(-a*x-b*(x**2))

popt, pcov = curve_fit(f, xdata, ydata, p0=[0.1, 1e-3])

print(popt)
```

Example of MCDS input and output files

Figure S1. Example of MCDS input file for 3.82 MeV α-particles.

mcds - Σημειωματάριο

Αρχείο Επεξεργασία Μορφή Προβολή Βοήθεια

```
!SAMPLE MCDS INPUT FILE (3.82 MeV alpha, 20% O2 concentration)

SIMCON: nocs=10000 seed=987654321
CELL: DNA=1  ndia=5
EVO2: pO2=20.0
RADX: par=4He ke=3.82
```

Figure S2. Example of MCDS output file, for 3.82 MeV α-particles.

Number of clusters per cell (DNA=1.000 Gbp, AD=1.000 Gy).

Number of	--------- DSB ----------		--------- SSB ----------		-------- OTHER ---------		----- ALL CLUSTERS -----	
lesions	Average	SEM	Average	SEM	Average	SEM	Average	SEM
1	---	---	1.95198E+01	1.70247E-02	5.86007E+01	2.83917E-02	7.81205E+01	3.23855E-02
2	1.78282E+00	5.39410E-03	2.21085E+01	1.80965E-02	3.08034E+01	2.13103E-02	5.46947E+01	2.78832E-02
3	2.93997E+00	6.87333E-03	1.91720E+01	1.72853E-02	1.61439E+01	1.55486E-02	3.82559E+01	2.38722E-02
4	3.24670E+00	7.11408E-03	1.50594E+01	1.53543E-02	8.47137E+00	1.13926E-02	2.67775E+01	2.01745E-02
5	3.08052E+00	7.00144E-03	1.11775E+01	1.31064E-02	4.44212E+00	8.18055E-03	1.87002E+01	1.64713E-02
6	2.68780E+00	6.55546E-03	8.07955E+00	1.12766E-02	2.32585E+00	6.02923E-03	1.30932E+01	1.40927E-02
7	2.24063E+00	5.90233E-03	5.69150E+00	9.50240E-03	1.21700E+00	4.42094E-03	9.14913E+00	1.16744E-02
8	1.80415E+00	5.29104E-03	3.96398E+00	7.97964E-03	6.43267E-01	3.29565E-03	6.41140E+00	9.88168E-03
9	1.40533E+00	4.74335E-03	2.72058E+00	6.57994E-03	3.30583E-01	2.31902E-03	4.45650E+00	8.38272E-03
10	1.09220E+00	4.14749E-03	1.85565E+00	5.38223E-03	1.77050E-01	1.70548E-03	3.12490E+00	6.85304E-03
11	8.32317E-01	3.64788E-03	1.25713E+00	4.48884E-03	9.28500E-02	1.25388E-03	2.18230E+00	5.78004E-03
12	6.26433E-01	3.22348E-03	8.46150E-01	3.72405E-03	4.61167E-02	8.76173E-04	1.51870E+00	4.92744E-03
13	4.72167E-01	2.77918E-03	5.77600E-01	3.06293E-03	2.58333E-02	6.51473E-04	1.07560E+00	4.16162E-03
14	3.48367E-01	2.38960E-03	3.85317E-01	2.54398E-03	1.26167E-02	4.64212E-04	7.46300E-01	3.48112E-03
15	2.54483E-01	2.04629E-03	2.57200E-01	2.03407E-03	6.76667E-03	3.34812E-04	5.18450E-01	2.88788E-03
16	1.88933E-01	1.75219E-03	1.72450E-01	1.68440E-03	3.76667E-03	2.49953E-04	3.65150E-01	2.43235E-03
17	1.37767E-01	1.50722E-03	1.15967E-01	1.37713E-03	2.16667E-03	1.90265E-04	2.55900E-01	2.04864E-03
18	1.01133E-01	1.29234E-03	7.49000E-02	1.11690E-03	1.00000E-03	1.28718E-04	1.77033E-01	1.69200E-03
19	7.19833E-02	1.09444E-03	5.19500E-02	9.25524E-04	4.00000E-04	8.15557E-05	1.24333E-01	1.42786E-03
20	5.19833E-02	9.34894E-04	3.40000E-02	7.45736E-04	2.00000E-04	5.77033E-05	8.61833E-02	1.19508E-03
21	3.72333E-02	7.84422E-04	2.24667E-02	6.13375E-04	2.66667E-04	6.66166E-05	5.99667E-02	1.00025E-03
22	2.58167E-02	6.50045E-04	1.48333E-02	5.03565E-04	5.00000E-05	2.88646E-05	4.07000E-02	8.24269E-04
23	1.96500E-02	5.71089E-04	8.93333E-03	3.83450E-04	5.00000E-05	2.88646E-05	2.86333E-02	6.82523E-04
24	1.40667E-02	4.85012E-04	7.18333E-03	3.43376E-04	1.66667E-05	1.66667E-05	2.12667E-02	5.90505E-04
25	9.43333E-03	3.97919E-04	4.31667E-03	2.69937E-04	3.33333E-05	2.35690E-05	1.37833E-02	4.84966E-04
26	6.80000E-03	3.34744E-04	2.78333E-03	2.16171E-04	1.66667E-05	1.66667E-05	9.60000E-03	3.99611E-04
27	5.10000E-03	2.88996E-04	1.96667E-03	1.79984E-04	---	---	7.06667E-03	3.38322E-04
28	3.88333E-03	2.54730E-04	1.20000E-03	1.40918E-04	---	---	5.08333E-03	2.91418E-04
29	2.60000E-03	2.06547E-04	1.03333E-03	1.30833E-04	---	---	3.63333E-03	2.43395E-04
30	1.78333E-03	1.74695E-04	7.00000E-04	1.07791E-04	---	---	2.48333E-03	2.06017E-04

31	1.40000E-03	1.52117E-04	2.83333E-04	6.86634E-05	---	---	1.68333E-03	1.66658E-04
32	9.50000E-04	1.25478E-04	2.50000E-04	6.45045E-05	---	---	1.20000E-03	1.40918E-04
33	7.00000E-04	1.07791E-04	2.00000E-04	5.77033E-05	---	---	9.00000E-04	1.22149E-04
34	4.00000E-04	8.15557E-05	1.00000E-04	4.08146E-05	---	---	5.00000E-04	9.11546E-05
35	4.16667E-04	8.32333E-05	1.66667E-05	1.66667E-05	---	---	4.33333E-04	8.48774E-05
36	1.50000E-04	4.99800E-05	8.33333E-05	3.72603E-05	---	---	2.33333E-04	6.23204E-05
37	1.66667E-04	5.26809E-05	6.66667E-05	3.33283E-05	---	---	2.33333E-04	6.23204E-05
38	2.33333E-04	6.23204E-05	5.00000E-05	2.88646E-05	---	---	2.83333E-04	6.86634E-05
39	3.33333E-05	2.35690E-05	1.66667E-05	1.66667E-05	---	---	5.00000E-05	2.88646E-05
40	3.33333E-05	2.35690E-05	---	---	---	---	3.33333E-05	2.35690E-05
41	5.00000E-05	2.88646E-05	---	---	---	---	5.00000E-05	2.88646E-05
42	5.00000E-05	2.88646E-05	1.66667E-05	1.66667E-05	---	---	6.66667E-05	3.33283E-05
43	3.33333E-05	2.35690E-05	---	---	---	---	3.33333E-05	2.35690E-05
44	1.66667E-05	1.66667E-05	---	---	---	---	1.66667E-05	1.66667E-05
45	1.66667E-05	1.66667E-05	---	---	---	---	1.66667E-05	1.66667E-05
46	1.66667E-05	1.66667E-05	---	---	---	---	1.66667E-05	1.66667E-05
47	---	---	---	---	---	---	---	---
48	---	---	---	---	---	---	---	---
49	---	---	---	---	---	---	---	---
50	---	---	---	---	---	---	---	---
Total	2.34967E+01	1.46931E-02	1.13188E+02	3.11692E-02	1.23347E+02	3.24383E-02	2.60032E+02	3.37061E-02

Example of WebPlotDigitizer software

We load the file and check 2D Plot. After that, we choose 2 points in X-axis (X1 and X2) and 2 points in Y-axis (Y1 and Y2) in order to define their values. For example, in the image below we choose X1=5, X2=10, Y1=40 and Y2=60. In that way, the values on axes are being adjusted correctly. In the case where an axis is on a logarithmic scale, we choose the Log Scale square.

Figure S3. Correct axis numbering.

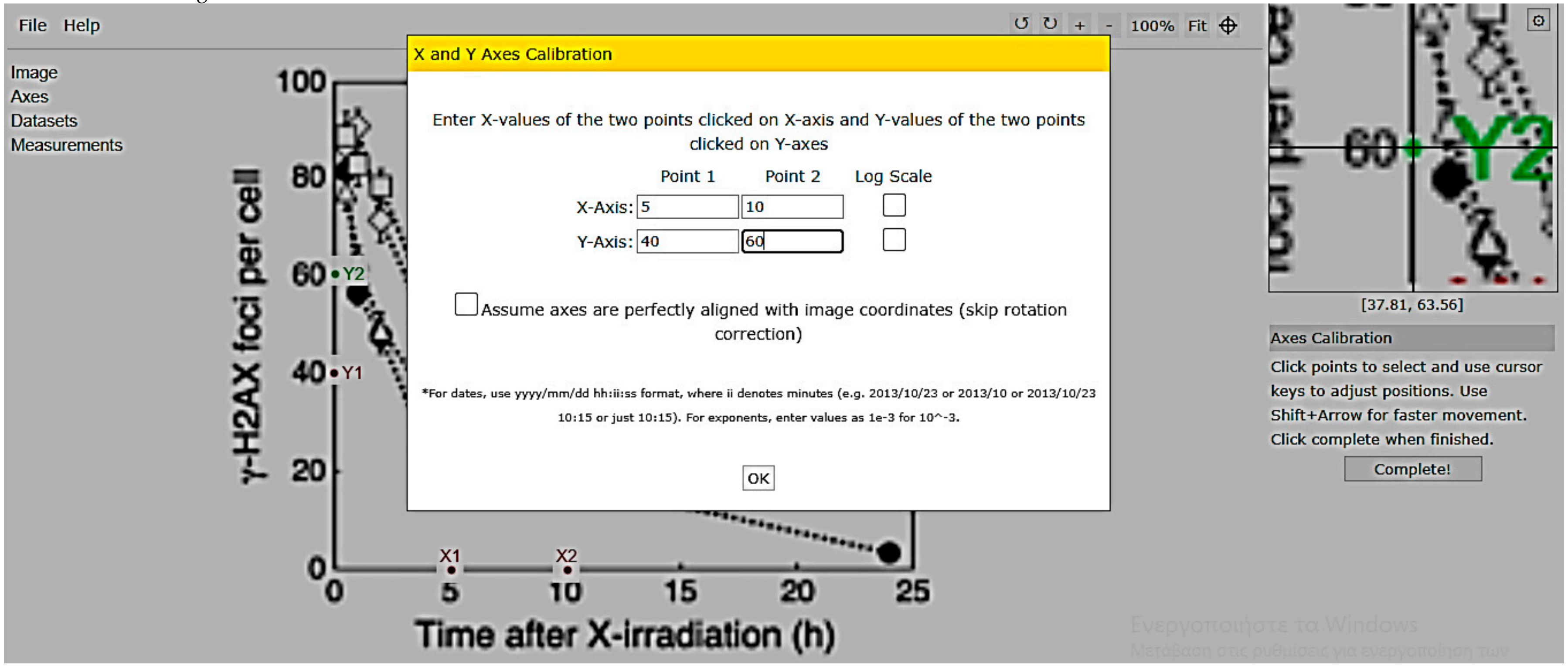

So, the diagram is ready. After that, we choose the experimental point which is of our interest and up in the right side of the image we can see the pair of its coordinates. We repeat this process for four-five different points, and we transfer the pair of coordinates into the code, in order to calculate α and β.

For example, below we choose with red, a point around five hours and 20 γH2Ax foci per cell. Then, we can see in detail its coordinates, in the right side of the image. More specifically, the x-value is 4.0733 and the y-value is 24.653. Finally we load this pair (4.0733, 24.653)

Figure S4. Selection of the appropriate point and the finding of its coordinates (Takanori Katsube, Masahiko Mori, Hideo Tsuji, Tadahiro Shiomi, Naoko Shiomi, Makoto Onoda, "Differences in sensitivity to DNA-damaging Agents between XRCC4- and Artemis-deficient human cells", 2011, Journal of Radiation Research).

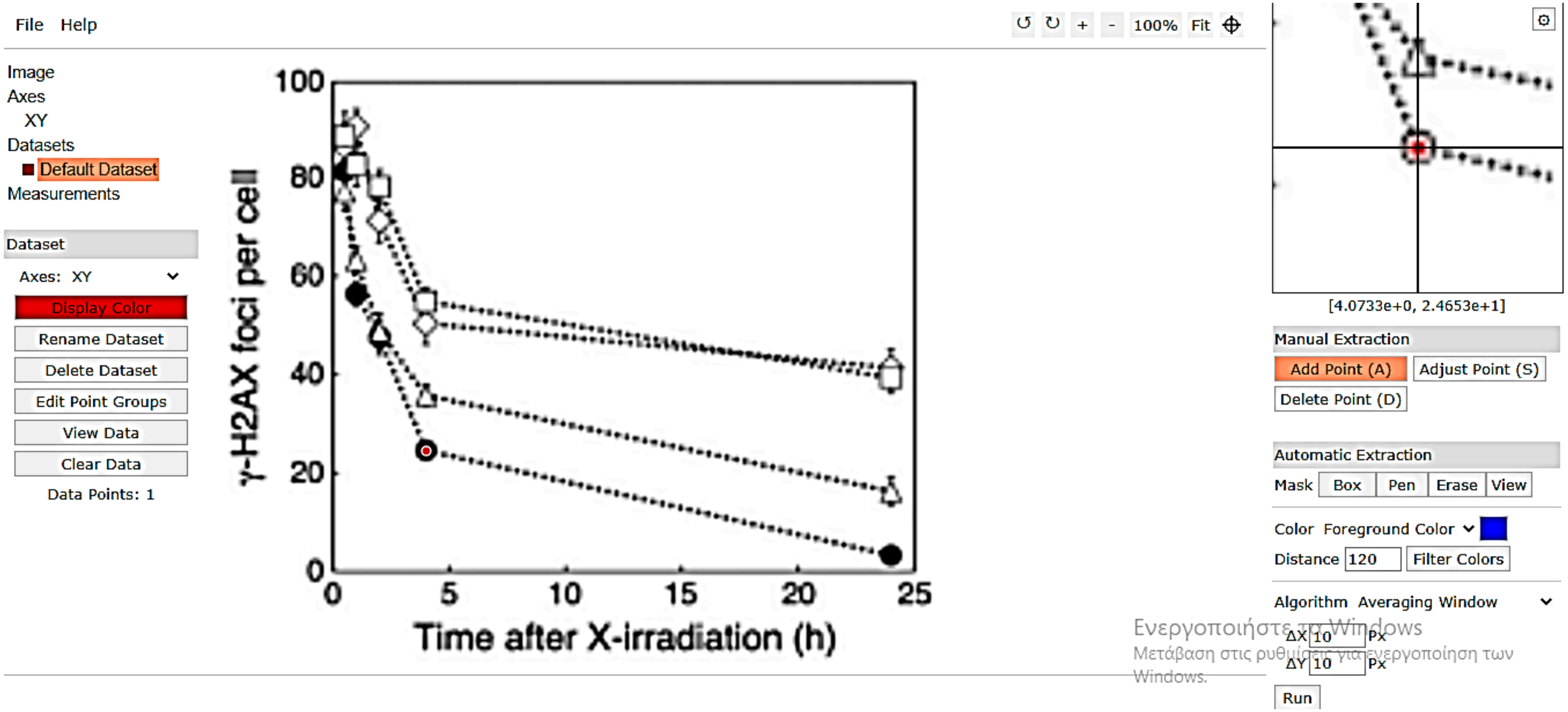

Table S1. X-ray repair data from literature and the k data from MATLAB calculations.

#ExpID	PMID	#Exp	CellLine	RadiationType	Tissue	CellClass	Energy (MeV)	LET (keV/μm)	Dose Rate (Gy/min)	DSBs/(Gy*cell)_0-0.15h	% DSBs_1h	% DSBs_2h	% DSBs_4h	% DSBs_12h	% DSBs_24h	DSBs_24h	**k of NHEJ**		
1	17644493	1	MRC-5	X-rays	fibroblast	n	0.120	2	1	42.624	75	40	21	7	1	0.426	Fibroblasts		
2	17644493	2	MRC-5	X-rays	fibroblast	n	0.025	2	0.5	33.92	75	42	26	8	0	0	**k1=27.665**	**k2=271.883**	**k3=5.278**
3	20197645	1	AT1OS/T-n	X-rays	fibroblast	n	0.150	2	1.3	13.952	90	82	70	38	33	4.604	**k4=4.935**	**k5=3.880**	**k6=5.106**
4	20197645	2	SuSa/T-n	X-rays	fibroblast	n	0.150	2	1.3	19.008	90	80	61	14	4	7.603	**k7=3.162**	**k8=1.511**	**k9=1.682**
5	20394838	1	AG1521	X-rays	fibroblast	n	N/A	N/A	0.9	20.544	80	61	42	28	20	4.109	**k10=0.065**		
6	19755805	1	HFFF2	X-rays	fibroblast	n	0.250	2	0.7	31.232	72	59	31	20	2	0.625			
7	17169382	1	VH25	X-rays	fibroblast	n	0.200	2	2.8	39.104	85	59	55	41	27	10.558			
8	17169382	2	FN1	X-rays	fibroblast	n	0.200	2	2.8	39.104	85	59	55	41	27	10.558			
9	18604166	1	MADA	X-rays	fibroblast	n	0.250	2	0.53	41.344	80	N/A	39	N/A	6	2.481			
10	18604166	2	K	X-rays	fibroblast	n	0.250	2	0.53	45.312	78	N/A	30	N/A	0	0			
11	26452569+	1	AG1522	X-rays	fibroblast	n	N/A	N/A	0.591	29.312	67	56	N/A	N/A	9	2.638			
12	24505255+	1	N/TERT-1	X-rays	fibroblast	n	N/A	N/A	0.591	17.6	50	41	24	15	1	0.176			
13	8618842+	1	GM38	X-rays	fibroblast	n	0.225	2	1.5	11.008	50	41	37	35	29	3.192			
14	22240940	1	He17	X-rays	fibroblast	n	N/A	N/A	0.635	23.232	88	64	46	25	11	2.556			
15	14744762	1	MRC-5	X-rays	fibroblast	n	0.09	2.5	2	24.896	N/A	49	24	N/A	5	1.245			
16	14744762	2	180BR	X-rays	fibroblast	n	0.09	2.5	2	36.928	N/A	64	62	N/A	28	10.340			
17	14744762	3	AT1BR	X-rays	fibroblast	n	0.09	2.5	2	22.656	N/A	43	38	N/A	21	4.758			

18	24312182	1	AG1522	X-rays	fibroblast	n	N/A	N/A	0.59	20.8	98	81	53	15	8	1.664			
19	18246480+	1	1BR3	X-rays	fibroblast	n	0.200	2	1.234	39.616	69	41	31	11	2	0.792			
20	18246480+	2	46BR	X-rays	fibroblast	n	0.200	2	1.234	37.248	75	52	27	11	6	2.235			
21	18246480+	3	180BR	X-rays	fibroblast	n	0.200	2	1.234	37.76	93	84	73	50	42	15.859			
22	30872656+	1	AG01522B	X-rays	fibroblast	n	N/A	N/A	N/A	24	61	41	31	15	9	2.16			
23	33692980+	1	Dental follicle stromal	X-rays	epithelial	n	0.120	2	0.015	32.448	92	77	49	N/A	17	5.516	Epithelial cells		
24	25040548	1	LN18	X-rays	epithelial	t	N/A	N/A	0.65	10.88	84	78	45	28	19	2.067	**k1=65.548**	**k2=11.873**	**k3=6.629**
25	25040548	2	U251	X-rays	epithelial	t	N/A	N/A	0.65	11.84	94	75	63	21	9	1.066	**k4=4.466**	**k5=4.633**	**k6=3.247**
26	21785230	1	HCT116	X-rays	epithelial	t	N/A	N/A	0.6	40.448	56	46	25	16	4	1.618	**k7=5.304**	**k8=13.840**	**k9=5.470**
27	26683123	1	MCF-7	X-rays	epithelial	t	N/A	N/A	0.59	19.904	89	88	79	66	53	10.549	**k10=0.062**		
28	24763056+	1	MiaPaCa2	X-rays	epithelial	t	0.200	2	1.3	21.056	83	63	46	20	7	1.474			
29	21785230	1	HCT116	X-rays	epithelial	t	N/A	N/A	0.6	40.96	69	58	29	18	1	0.410			
30	26991853	1	H460	X-rays	epithelial	t	0.160	2	2.5	24.64	87	75	57	31	13	3.203			
31	32397297+	1	A549	X-rays	epithelial	t	0.200	2	0.85	19.328	88	81	61	21	4	0.773			
32	22322361	1	PBMCs	X-rays	blood	n	0.250	2	0.26	13.888	89	73	43	39	29	4.028	Lymphocytes		
33	20597840	1	T-lymphocytes	X-rays	blood	n	N/A	N/A	0.02	12.736	96	N/A	N/A	N/A	39	4.967	**k1=43.885**	**k2=152.894**	**k3=4.839**
34	20597840	2	T-lymphocytes	X-rays	blood	n	N/A	N/A	0.02	14.72	97	N/A	N/A	N/A	0	0	**k4=4.450**	**k5=3.079**	**k6=4.066**
35	22144029	1	Lymphocytes	X-rays	blood	n	N/A	N/A	N/A	6.336	65	55	N/A	N/A	22	1.394	**k7=2.642**	**k8=1.896**	**k9=2.053**

																	**k10=0.039**		

This model is formulated below by a system of 9 nonlinear ODEs: (Reza Taleei, Hooshang Nikjoo, "The Non-homologous End-Joining (NHEJ) Pathway for the Repair of DNA Double-Strand Breaks: I. A Mathematical Model", RADIATION RESEARCH **179** (2013))

$$\frac{dy_1}{dt} = \frac{a}{C}\cdot\frac{dD}{dt} - v_1,\ \frac{dy_2}{dt} = v_1 - v_2,\ \frac{dy_3}{dt} = v_2 - v_3,\ \frac{dy_4}{dt} = v_3 - v_4,$$
$$\frac{dy_5}{dt} = v_4 - v_5 - v_7,\ \frac{dy_6}{dt} = v_5 - v_6,\ \frac{dy_7}{dt} = v_7 - v_8,\ \frac{dy_8}{dt} = v_8 - v_9$$
$$\frac{dy_9}{dt} = v_9 - v_{10}$$

Where

$$v_1 = k_1(1-c_2)y_1,\ v_2 = k_2(1-c_3)y_2,\ v_3 = k_3y_3,\ v_4 = k_4y_4,$$
$$v_5 = k_5(1-c_6)y_5,\ v_6 = K_6y_6,\ v_7 = k_7(1-c_7)y_5,\ v_8 = k_8(1-c_8)y_7,$$
$$v_9 = k_9(1-c_9)y_8,\ v_{10} = K_{10}y_9$$

The scaling factor C is the sum of repair proteins (E_i) and repair complex concentrations (Y_i):

$$C = \sum_{i=1}^{8} E_i + \sum_{i=1}^{8} Y_i = cte = 3000,\ y_i = \frac{Y_i}{C},\ c_i = \frac{\sum_{j=i}^{8} y_j}{C}$$

The variables y_i, v_i, k_i are the scaled repair complex, repair rate, and repair rate constant for i = 1 to 9, respectively.

In the tables and figures below, the experimental data are the calculated repair data from Table S1, while theoretical values are the fitted ones from the NHEJ model simulation.

Table S2. Experimental and theoretical values for epithelial cells.

Time (h)	Deq (Gy) (Exp)	Deq (Gy) (Theor)
1	82	68
2	68	51
4	47	44
12	28	27
24	14	13

Figure S5. Fitting epithelial data with the NHEJ model.

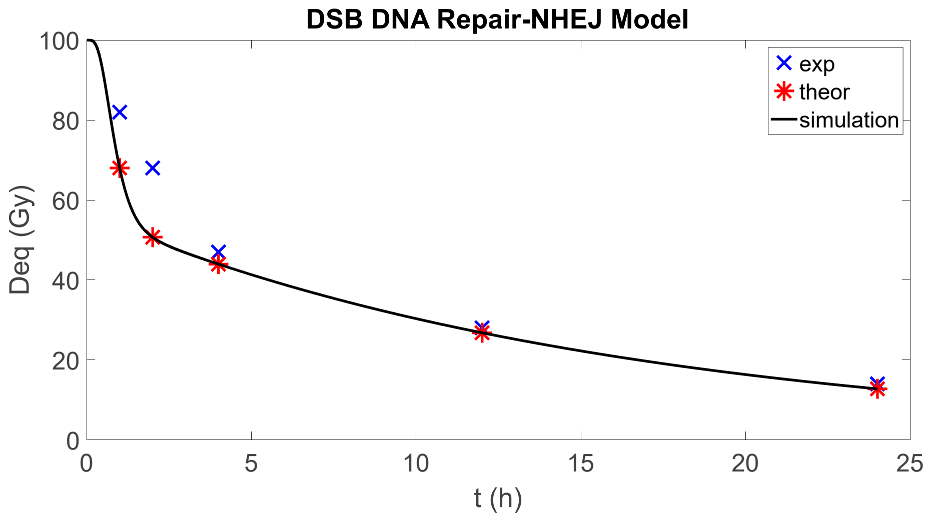

Table S3. Experimental and theoretical values for lymphocytes.

Time (h)	Deq (Gy) (Exp)	Deq (Gy) (Theor)
1	84	64
2	64	46
4	46	42
12	28	31
24	23	20

Figure S6. Fitting lymphocyte data with the NHEJ model.

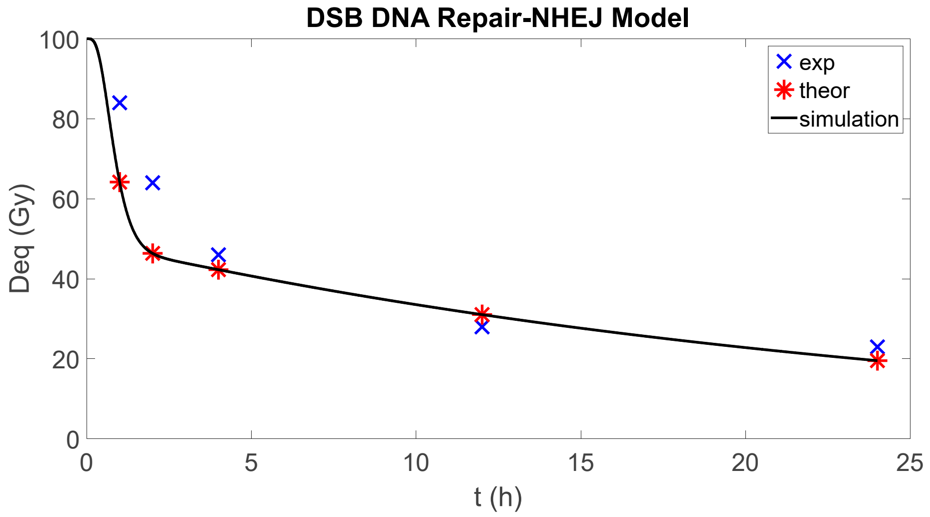

Table S4. Experimental and theoretical values for fibroblasts.

Time (h)	Deq (Gy) (Exp)	Deq (Gy) (Theor)
1	77	58
2	57	44
4	42	39
12	23	23
24	13	11

Figure S7. Fitting fibroblast data with the NHEJ model.

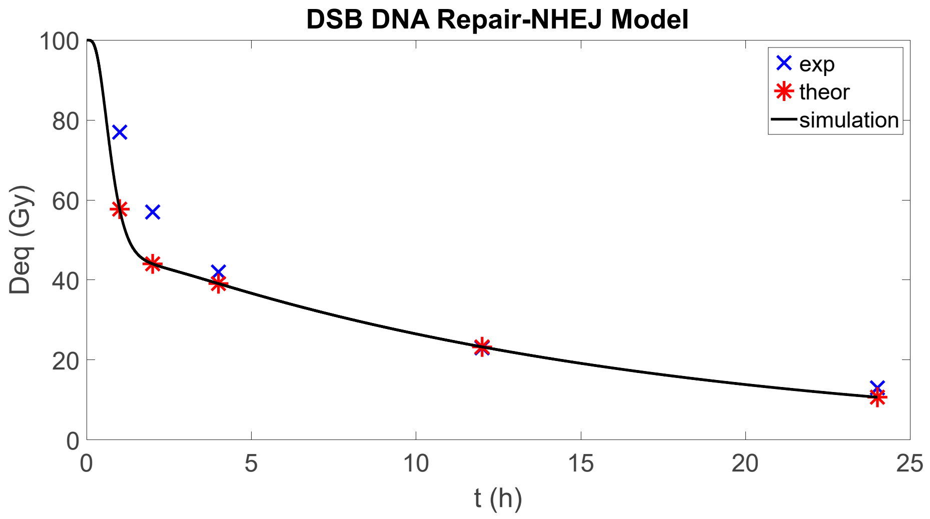

MATLAB code for DNA repair

```
function dy=myodes(t,y,k)

C=3000;
a=0.2;
dDdt=80;

% ***********************************
% Constants for Model
%k1=350;k2=500;k3=50;k4=20;k5=15;
%k6=5;k7=3.6;k8=8;k9=0.25;k10=0.55;

% k1=100;k2=300;k3=5;k4=2;k5=1;
% k6=5;k7=1;k8=1;k9=0.25;k10=0.05;

% ****************************************


y1=y(1);y2=y(2);y3=y(3);y4=y(4);y5=y(5);y6=y(6);y7=y(7);y8=y(8);y9=y(9);
k1=k(1);k2=k(2);k3=k(3);k4=k(4);k5=k(5);k6=k(6);k7=k(7);k8=k(8);k9=k(9);k10=k(10);

c1=sum(y)/C;c2=sum(y(2:end))/C;c3=sum(y(3:end))/C;c4=sum(y(4:end))/C;
c5=sum(y(5:end))/C;c6=sum(y(6:end))/C;c7=sum(y(7:end))/C;c8=sum(y(8:end))/C;
c9=y(9)/C;

v1=k1*(1-c2)*y1;v2=k2*(1-c3)*y2;v3=k3*y3;v4=k4*y4;
v5=k5*(1-c6)*y5;v6=k6*y6;v7=k7*(1-c7)*y5;v8=k8*(1-c8)*y7;
v9=k9*(1-c9)*y8;v10=k10*y9;

dy(1)=a*dDdt/C-v1;dy(2)=v1-v2;dy(3)=v2-v3;dy(4)=v3-v4;
dy(5)=v4-v5-v7;dy(6)=v5-v6;dy(7)=v7-v8;dy(8)=v8-v9;dy(9)=v9-v10;
dy=dy';


% Program for fitting ODEs to data
clc
clear
close all

% The data points
```

```
Data=xlsread('DSB DATA.xlsx',6);
exp_t=Data(:,1);
exp_Deq=Data(:,2:end);
%exp_Deq2=Data(:,3);
%k0=[100;300;5;2;1;5;1;1;0.25;0.05]; % Initial guess for k parameters
%k0=[100;300;5;2;1;5;1;1;0.06;0.05];
k0=[100*rand;400*rand;5*rand;4*rand;4*rand;5*rand;2*rand;2*rand;0.7*rand;0.1*rand];

% Finding the fit parameters

%first method with fitnlm
%mdl = fitnlm(exp_t,exp_Deq,@SSA,k0)

% Second method with lsqnonlin
fun=@(k)SSA(k,exp_t)-exp_Deq;
opts = optimoptions(@lsqnonlin,'Algorithm','levenberg-marquardt');
k=lsqnonlin(fun,k0,[],[],opts);


% Solving the ODEs for the estimated parameters
tspan=[0 24];
y0=[100;0;0;0;0;0;0;0;0]; % y01=initial unrepaired cells
options = odeset('RelTol',1e-8,'AbsTol',1e-10);
[time,Y] = ode45(@(t,y)myodes(t,y,k),tspan,y0,options);
Deq=sum(Y,2);

% Making the plot of solutions and corresponding experimental data

h=plot(exp_t,exp_Deq,'x',time,Deq,'linewidth',3);
set(h,'Markersize',22)
set(gca,'fontsize',28)
xlabel('t (h)')
ylabel('Deq (Gy)')
legend(h([1 9]),'exp','model')
xlim([0 24])
ylim([0 100])
```

```
box on
grid on
display(k')

function C=SSA(k,t)
y0=[100;0;0;0;0;0;0;0;0];
options = odeset('RelTol',1e-8,'AbsTol',1e-10);
%[T,Yv]=ode45(@(t,y)myodes(t,y,k),t,y0,options);
[T,Yv]=ode23tb(@(t,y)myodes(t,y,k),t,y0,options);

C=sum(Yv,2);
end
```

```
% This code is for DSB DNA repair described by NHEJ model
clc
clear
close all

% Experimental Data
Data=xlsread('DSB DATA.xlsx',7);
exp_t=Data(:,1);
exp_Deq=Data(:,2);

% Solving the system of ODEs
tspan=[0 24]; % Time Interval in hours
y0=[100;0;0;0;0;0;0;0;0]; % initial unrepaired cells
k0=[27.6652;271.8827;5.2783;4.9348;3.8797;5.1055;3.1620;1.5105;1.6822;0.0652];
options = odeset('RelTol',1e-8,'AbsTol',1e-10);
[t,y]=ode45(@(t,y,k)myodes(t,y,k0),tspan,y0,options);

% The sum of all yi for every value of time (row addition of y-values)
 Deq=sum(y,2);

 % Find theoretical value of Deq in certain time values
 t1=[1;2;4;12;24];
```

```
Deqth=interp1(t,Deq,t1,'spline');
disp(Deqth)


%Graph the solution
h=plot(exp_t,exp_Deq,'bx',t1,Deqth,'r*',t,Deq,'k','linewidth',3);
xlabel('t (h)');ylabel('Deq (Gy)');
title('DSB DNA Repair-NHEJ Model')
set(h,'Markersize',22) %,'MarkerFaceColor','r')
set(gca,'fontsize',28)
ylim([0 100])
legend('exp','theor','simulation')
box on
grid on
```

Useful information on the development of the Database and ML model :

- CODE for the creation of database in GitHub: https://github.com/gokaybiz/radphysbio and also https://github.com/gokaybiz/radphysbio/tree/data
- CODE of the ML model in GitHub: https://github.com/dimitrispapak/multioutput_regression/blob/main/multioutput.R

Tuning of ML algorithm

Figure S8. Error evolution during training in dependence with the amount of trees in the random forest. The final model is trained with 100 trees.

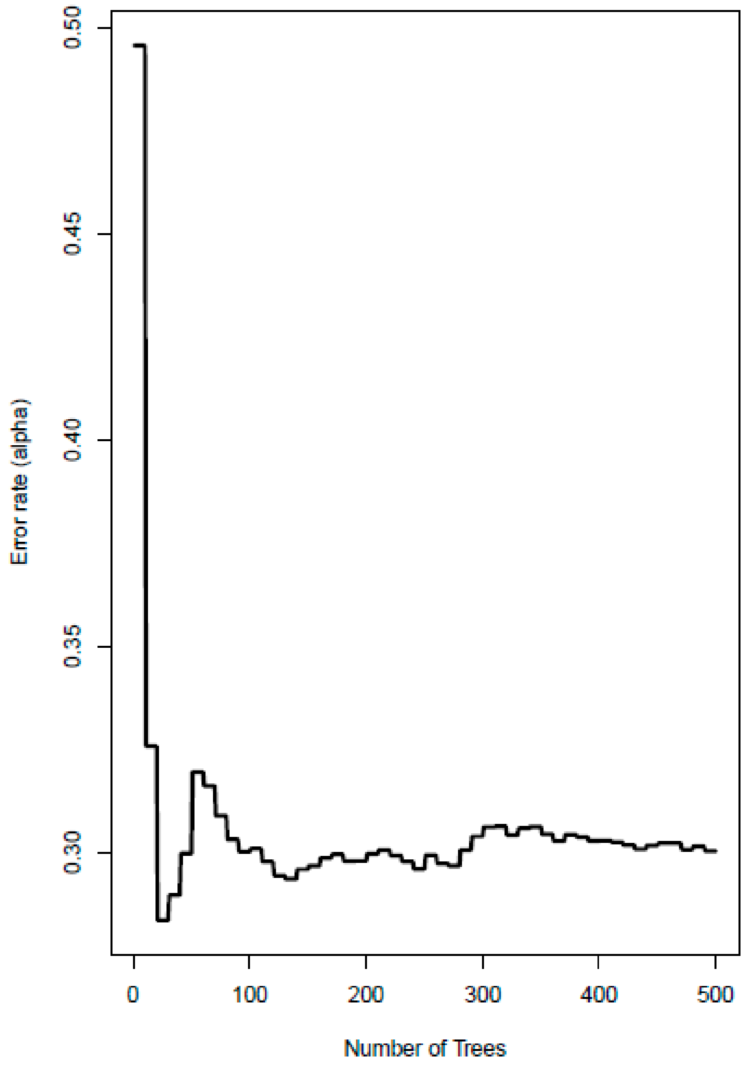

ML results, actual versus predicted, where training-testing set are split in ratio 70% - 30%

Figure S9. Plots of the distribution of α-values versus the distribution of their predictions, including the calculation of Spearman correlation coefficient: (**a**) α performance, (**b**) α performance (0-2 Gy^{-1}), (**c**) logα performance.

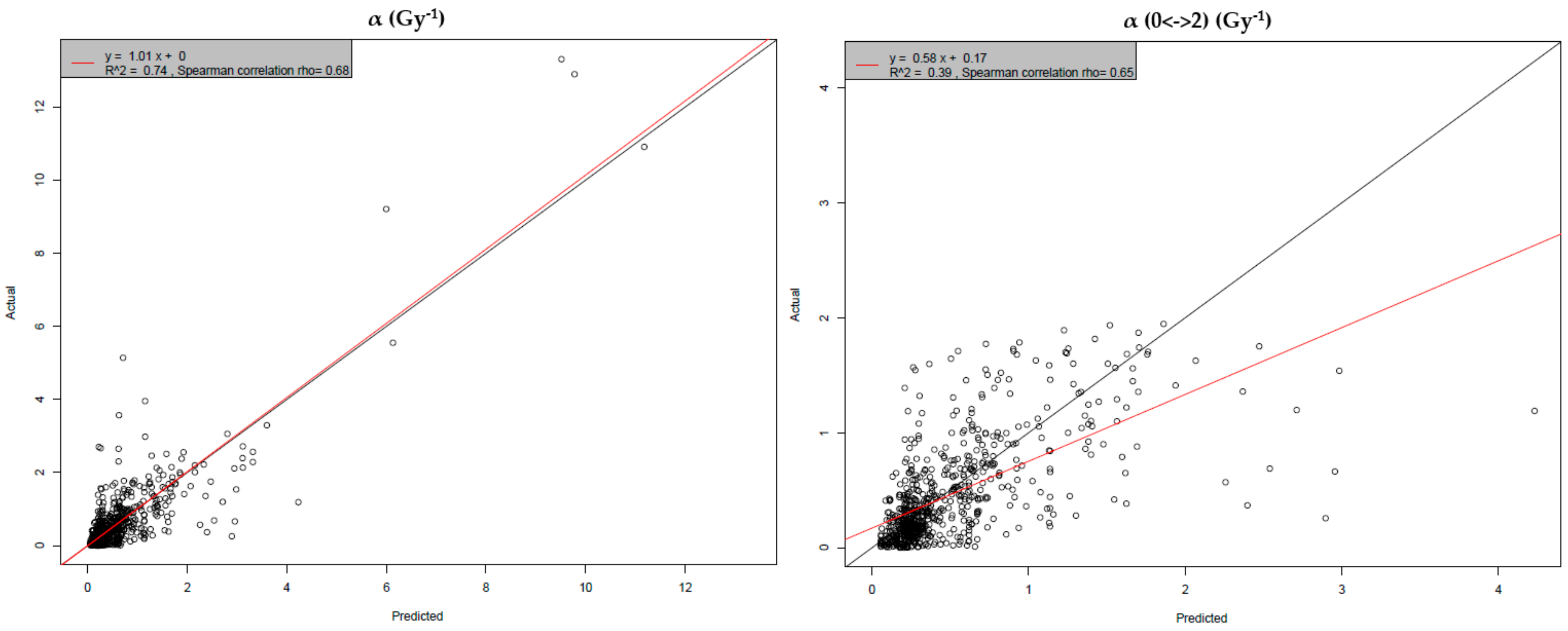

α (Gy-1)
y = 1.01 x + 0
R^2 = 0.74 , Spearman correlation rho= 0.68
Actual
Predicted
α (0<->2) (Gy-1)
y = 0.58 x + 0.17
R^2 = 0.39 , Spearman correlation rho= 0.65
Actual
Predicted

(**a**) (**b**)

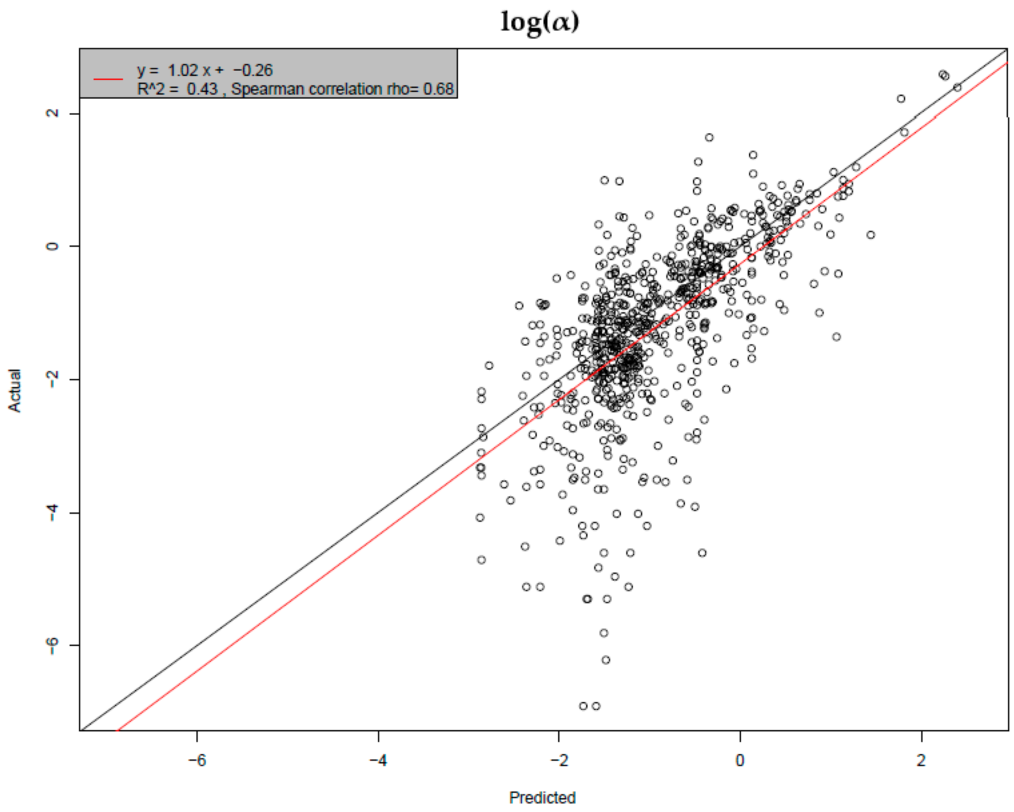
log(α)
y = 1.02 x + −0.26
R^2 = 0.43 , Spearman correlation rho= 0.68
Actual
Predicted
2
0
−2
−4
−6
−6
−4
−2
0
2

(c)

Figure S10. Plots of the distribution of β-values versus the distribution of their predictions, including the calculation of Spearman correlation coefficient: (**a**) β performance, (**b**) β performance (0-2 Gy-2).

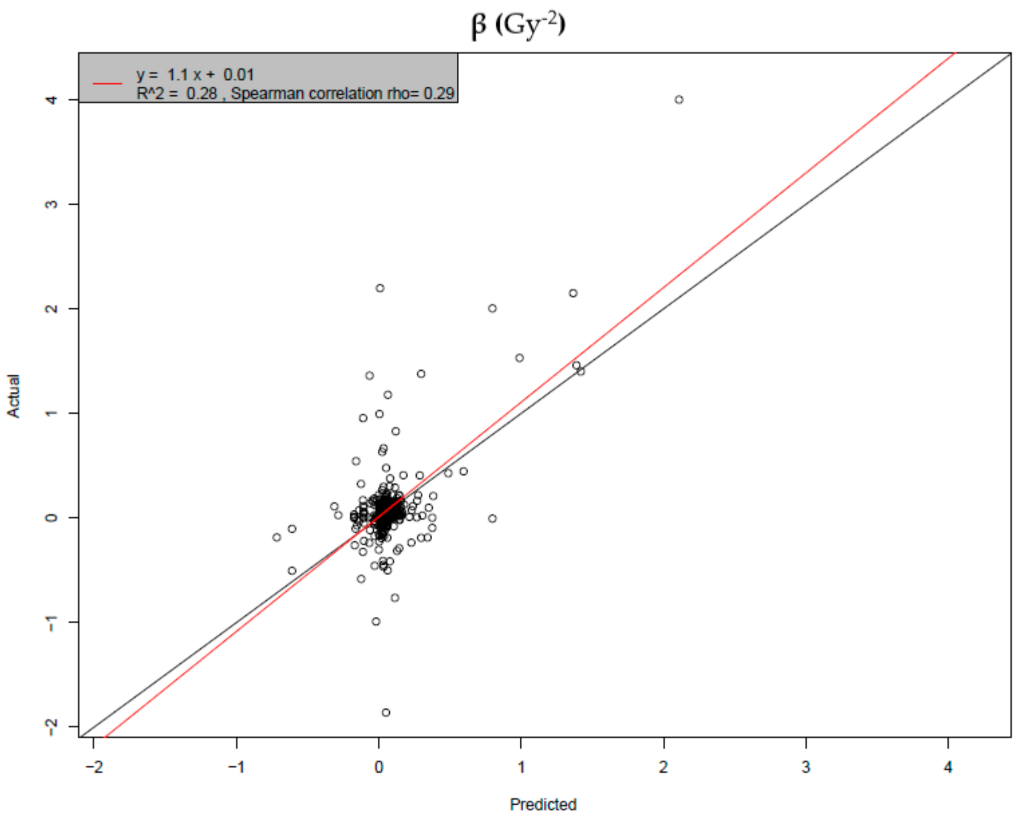

(**a**)

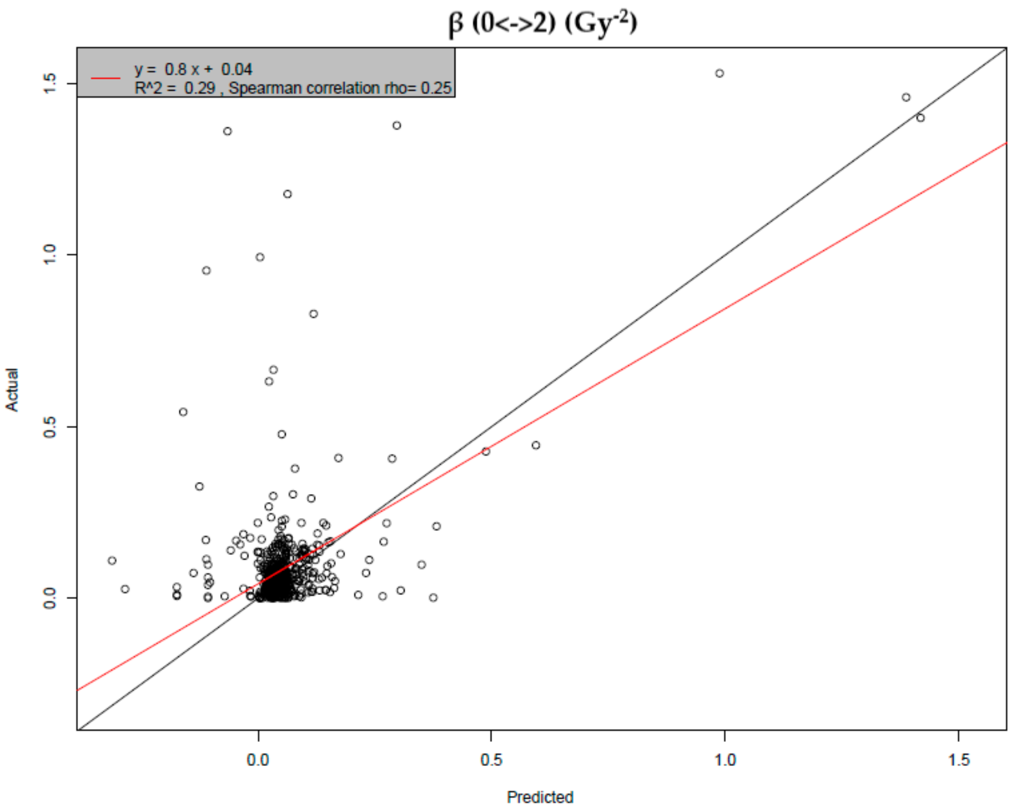

(**b**)

Table S5. Presentation of the values of Mean absolute error, RMSE and Spearman correlation coefficient for: α, α (0-2 Gy-1), β and β (0-2 Gy-2).

	alpha	alpha 0 <-> 2	beta	beta 0 <-> 2
**Mean Absolute Error**	0.293	0.245	0.094	0.061
**RMSE**	0.54	0.395	0.272	0.144
**Spearman Rho**	0.681	0.65	0.292	0.245
**R^2**	0.735	0.387	0.276	0.288

Figure S11. Permutation variable importance. The effect of each variable on the Out-Of-Bag (OOB) error during model training for (**a**) α and (**b**) β.

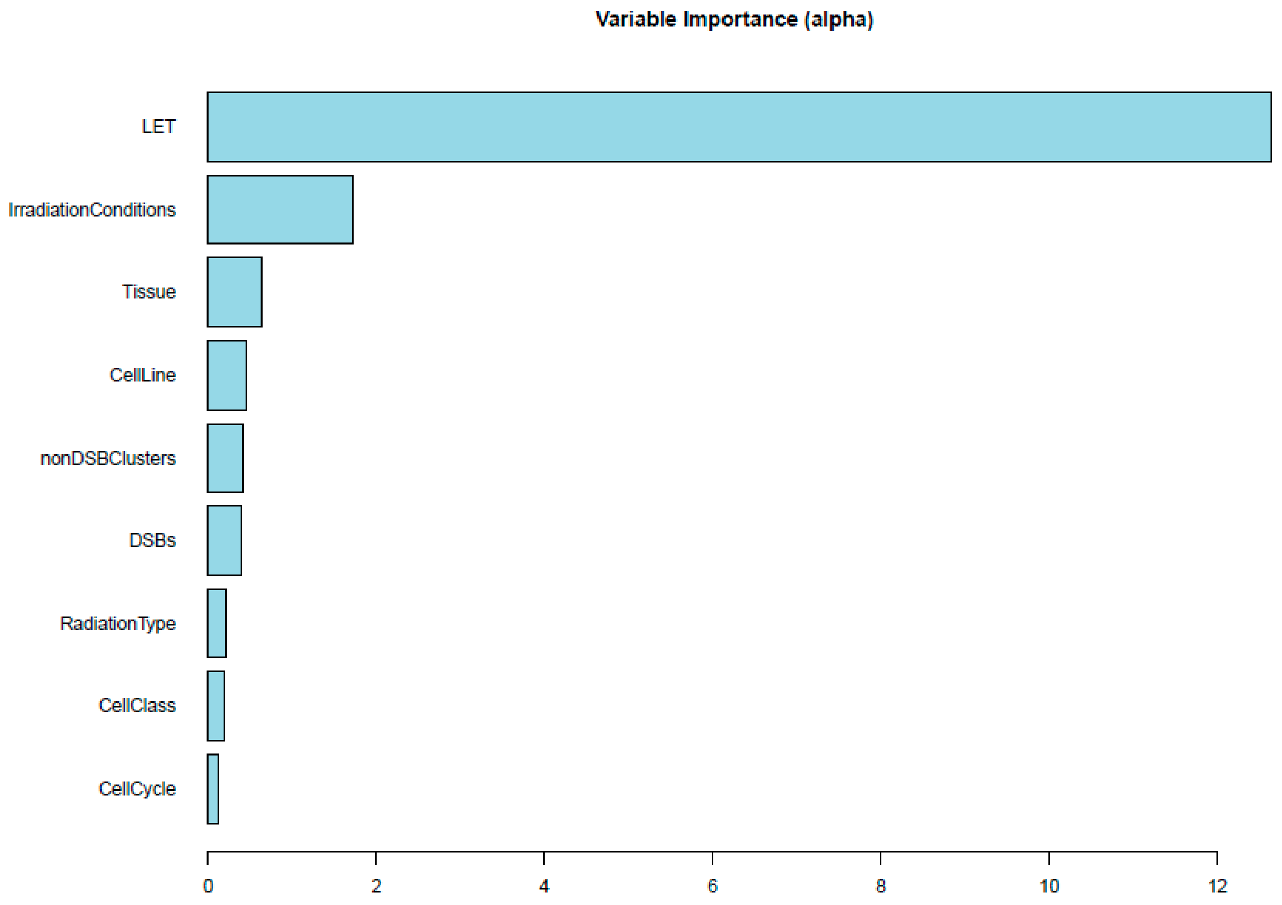

(**a**)

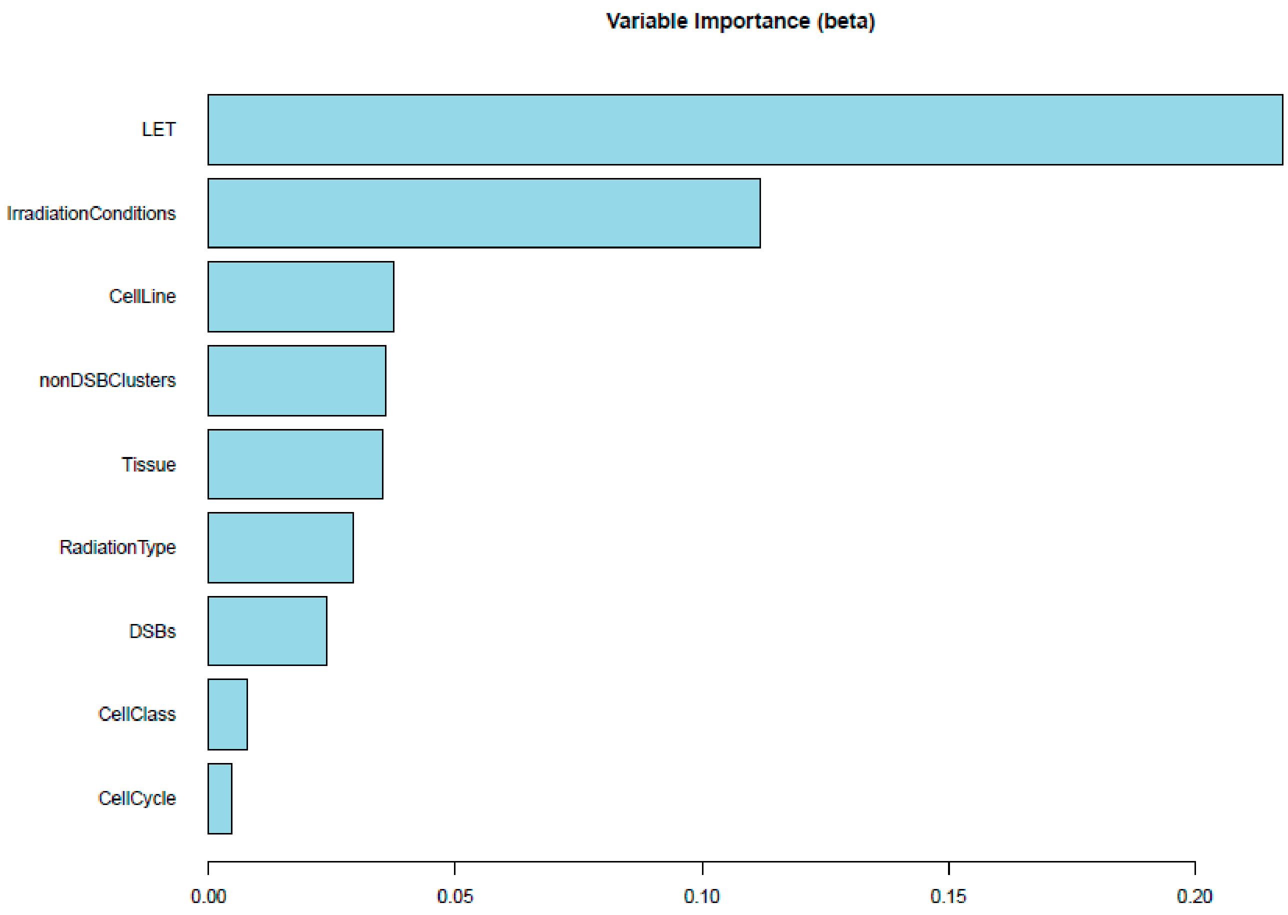
Variable Importance (beta)
LET
IrradiationConditions
CellLine
nonDSBClusters
Tissue
RadiationType
DSBs
CellClass
CellCycle
0.00
0.05
0.10
0.15
0.20

(**b**)
